# A New Player in the Development of TRAIL Based Therapies for Hepatocarcinoma Treatment: ATM Kinase

**DOI:** 10.3390/cancers4020354

**Published:** 2012-04-05

**Authors:** Venturina Stagni, Simonetta Santini, Daniela Barilà

**Affiliations:** 1 Department of Biology, University of Tor Vergata, Rome 00133, Italy; E-Mails: venturina.stagni@gmail.com (V.S.); santini.simonetta80@gmail.com (S.S.); 2 Laboratory of Cell Signaling, Santa Lucia Foundation-IRCCS, Rome 00179, Italy

**Keywords:** ATM kinase, hepatocellular carcinoma, TRAIL, combined therapy

## Abstract

Hepatocellular carcinoma (HCC) is one of the most common cancers worldwide. HCCs are genetically and phenotypically heterogeneous tumors characterized by very poor prognosis, mainly due to the lack, at present, of effective therapeutic options, as these tumors are rarely suitable for radiotherapy and often resistant to chemotherapy protocols. In the last years, agonists targeting the Tumor Necrosis Factor Related Apoptosis Inducing Ligand (TRAIL) death receptor, has been investigated as a valuable promise for cancer therapy, based on their selectivity for malignant cells and low toxicity for healthy cells. However, many cancer models display resistance to death receptor induced apoptosis, pointing to the requirement for the development of combined therapeutic approaches aimed to selectively sensitize cancer cells to TRAIL. Recently, we identified ATM kinase as a novel modulator of the ability of chemotherapeutic agents to enhance TRAIL sensitivity. Here, we review the biological determinants of HCC responsiveness to TRAIL and provide an exhaustive and updated analysis of the molecular mechanisms exploited for combined therapy in this context. The role of ATM kinase as potential novel predictive biomarker for combined therapeutic approaches based on TRAIL and chemotherapeutic drugs will be closely discussed.

## 1. Role of Apoptosis in Cancer

Apoptosis is an essential process to preserve tissue homeostasis and ensure the balance between cell loss and cell gain in normal tissue. Two main pathways trigger apoptosis: (i) the extrinsic pathway elicited by death receptor stimulation and (ii) the intrinsic pathway elicited in response to cellular stresses such as DNA damage and oxidative stress. The regulation of the apoptotic response in the liver mainly relies on the activation of death receptors, including Fas/CD95/APOI, Tumor Necrosis Factor (TNF) receptor 1 and the Tumor necrosis factor Related Apoptosis Inducing Ligand (TRAIL) receptors. In general, upon the binding of the corresponding ligands, the death receptors oligomerize and allow the assembly of the Death Inducing Signaling Complex (DISC) by recruiting the adaptor protein FADD and the initiator caspases, caspase-8 and caspase-10. Caspase-8 and caspase-10 do not heterodimerize at the same DISC complex but may both be independently recruited to different DISCs and initiate the apoptotic cascade. In any case, their recruitment at the DISC allows their autocatalytic processing, activation and release in the cytoplasm of caspase-8 and -10, which in turn trigger the activation of the downstream caspases, such as caspase-3, -6 and -7. Engagement of the extrinsic pathway by death receptors is sufficient in some cell types to trigger apoptosis, whereas in others amplification of this pathway through the activation of the intrinsic pathway is needed to commit the cell to apoptosis. The intrinsic pathway is mainly elicited in response to stress signals such as DNA damage and oxidative stress, which directly impinge on mitochondrial functionality leading to dissipation of the mitochondrial transmembrane potential, cytocrome C release to the cytoplasm, assembly of the apoptosome, activation of the initiator caspase-9 which in turn triggers the activation of the downstream effector caspases. The crosstalk between the extrinsic and intrinsic pathways relies on the cleavage and translocation of Bcl-2 family members, such as BID, to the mitochondria, in response to death receptor stimulation. This event allows the primer of the intrinsic signalling pathway and the amplification of the apoptotic signalling cascade (reviewed in [1]). The deregulation of apoptosis is a principal mechanism contributing to cancer development in many tissues, including liver, as well as to cancer resistance to therapies. The apoptotic response in the liver is also modulated in response to hepatitis viral infection as well as in other contexts of liver injury (reviewed in [2]).

## 2. Role of TRAIL Signalling in HCC Development and Therapy

TRAIL was identified through a screen of the human genome database for homologues to TNF. Indeed it shows 19% and 23% sequence identity respectively to TNF and Fas. TRAIL can interact with five different receptors (reviewed in [3]). However, it triggers apoptosis via two of its receptors, TRAIL-R1 (DR4) and TRAIL-R2 (DR5). Upon binding of TRAIL or agonistic antibodies to TRAIL-R1 or TRAIL-R2, the DISC is formed as described above, caspase-8 and -10 are activated and may drive the subsequent execution of the apoptotic response through mitochondrial independent and mitochondrial dependent pathways (reviewed in [3]).

In mice, there is only 1 apoptosis-inducing receptor for TRAIL, named TRAIL-R, which is equally related to human TRAIL-R1 and TRAIL-R2 [4]. TRAIL [5] and TRAIL-R [6] knockout mice have been generated and their susceptibility to cancer and to metastasis development has been investigated. Although the results are not always consistent among the different studies, the loss of function of these molecules, in agreement with their relevant role in the control of the apoptotic response, seems to promote to certain extent, inflammation and tumor initiation and metastasis. TRAIL-R^−/−^ mice were challenged with the hepatocarcinogen diethylnitrosamine (DEN) in a complete carcinogen protocol. A slightly increased incidence of HCC in DEN-injected TRAIL-R^−/−^ mice as compared to wt mice was observed. Furthermore, immunohistochemical analysis showed a reduction in the presence of apoptotic (TUNEL-positive) areas in HCCs from TRAIL-R^−/−^ compared to wt animals, suggesting a block of apoptosis [7].

Overall these studies support an essential role of TRAIL signalling in the control of cellular homeostasis and in the prevention of cancer development. Therefore the TRAIL pathway acts as a natural component of the endogenous tumor-surveillance system in mammals. Interestingly, when exogenously administered, TRAIL exerts a potent tumoricidal activity on cancer cells *in vitro* and *in vivo*, without significant effects on normal cells, pointing to its strong therapeutic potential [8].

At present three potential pharmacological strategies based on TRAIL signalling targeting have been designed: (i) use of recombinant human TRAIL; (ii) administration of activating humanized antibodies directed against TRAIL receptors TRAIL-R1 or TRAIL-R2; (iii) adenoviral delivery of the TRAIL coding sequence into tumor cells (Ad5-TRAIL). These compounds have been largely tested *in vitro* on several cancer cell lines, including HCC cells. Early *in vitro* studies raised concerns regarding the potential toxicity of TRAIL towards normal hepatocytes [9]. However, later reports suggest that the toxicity may rise from the use of tagged-TRAIL (histidine, FLAG or leucine-tagged TRAIL), while the recombinant untagged version of this cytokine may not be toxic [10]. Furthermore, in some circumstances, although the tagged-version of TRAIL show toxicity on primary hepatocytes and on hepatic explants from patients with impaired liver function, the same compounds were largely safe on hepatic explants obtained from healthy donors, supporting the potentiality of TRAIL targeting for HCC cancer therapy although great caution in the administration of TRAIL to patients with impaired liver function must be taken into account [11]. Therefore multiple clinical trials have been and are being conducted in order to define TRAIL therapeutic potential. A complete list of the compounds used in these trials has been provided in Table 1. Reports from phase I and phase II clinical trials conducted so far using these compounds clarified that contrary to what observed in some in vitro experiments, hepatic and renal toxicity were not generally detected and in any case were not clinically significant. Furthermore, no immunogenicity against humanized antibodies specific for TRAIL receptors TRAIL-R1 or TRAIL-R2 has been observed. Importantly, studies including patients with liver tumors, showed significant clinical responses, ranging from a partial response to even a complete response, observed using untagged version of recombinant human TRAIL or antibodies targeting TRAIL-R1 or TRAIL-R2 as monotherapy [12]. Overall these studies encourage the use of these agents for cancer therapy. However, clinical trials have also highlighted that several human tumors might be resistant to TRAIL, revealing the presence also *in vivo* of several molecular mechanisms that may trigger TRAIL resistance and point to their identification and their targeting as a valuable tool to develop TRAIL based combined therapy approaches aimed to augment TRAIL sensitivity *in vitro* and *in vivo*.

**Table 1:** TRAIL targeting ompounds.

TRAIL	Description	Company	Reference/Phase
Apo2L/TRAIL (AMG 951)	Soluble TRAIL activates TRAIL-R1 and TRAIL-R2 (DR4 and DR5)	Amgen/Genentech	Phase I/II Ongoing trial active, not recruiting.
HGS-ETR1 (Mapatumumab)	Humanized anti-TRAIL-R1 (DR4) agonistic mAb	Human Genome Science	Completed [13,14] More trials currently active or recruiting.
HGS-ETR2 (Lexatumumab)	Humanized anti-TRAIL-R2 (DR5) agonistic mAb	Human Genome Science	Phase I advanced solid tumors. Completed [12,15], More trials currently active or recruiting.
HGS-TR2J	Humanized anti-TRAIL-R2 (DR5) agonistic mAb	Human Genome Science	Phase I No ongoing trials [16].
TRA-8 (CS-1008; Tigatuzumab)	Humanized anti-TRAIL-R2 (DR5) agonistic mAb	Daiiki Sankyo Inc.	More trials currently recruiting [17,18].
Conatumumab (AMG 655)	Humanized anti-TRAIL-R2 (DR5) agonistic mAb	Amgen/Takeda	More trials currently active [19,20].
Apomab	Humanized anti-TRAIL-R2 (DR5) agonistic mAb	Genentech	No ongoing trials [21].
LBY135	Chimeric anti-TRAIL-R2 (DR5) agonistic mAb	Novartis	Phase I/II: advanced solid tumors No ongoing trials [22].

## 3. Molecular Mechanisms That Trigger TRAIL Resistance in HCC

HCC cells constitutively express TRAIL mRNA and protein, although there are contradictory reports about the expression of TRAIL receptors. The observation that most of HCC cells are insensitive towards TRAIL-induced apoptosis, point to the presence in HCC of several molecular mechanisms that trigger apoptosis resistance in general and more specifically TRAIL resistance (reviewed in [23]).

### 3.1. Role of the Expression of the Different TRAIL Receptors

TRAIL-R1 and TRAIL-R2 mRNA are widely expressed in normal tissues, while the expression of the corresponding proteins appears to be more restricted to infected, malignant or damaged cells. Cell surface levels of TRAIL-R1 and TRAIL-R2 are not clearly correlated to the cellular sensitivity to TRAIL although agents that increase their expression have been shown to enhance TRAIL sensitivity [24]. Interestingly, the chromosomal region where TRAIL-R1 and TRAIL-R2 are located undergoes hemizygous deletion in certain cancers and epigenetic silencing of TRAIL-R1 has been found in many tumors (reviewed in [25]). More interestingly, post translational modifications have been suggested to modulate TRAIL receptor signalling. *O*-glycosylation of TRAIL-R1 and TRAIL-R2 has been shown to enhance ligand dependent receptor clustering, necessary for the initiation of the apoptotic signalling [24]. Indeed the expression levels of a panel of O-glycosyltransferases is directly proportional to TRAIL sensitivity in a variety of cancer cell lines [24].

One important issue in the modulation of TRAIL-R1 and TRAIL-R2 functionality is the expression of three additional receptors for TRAIL in humans, named DcR1, DcR2 and osteoprotegrin (OPG). These are decoy receptors, which are able to bind TRAIL but are unable to transmit downstream apoptotic signalling. Therefore, decoy receptors, compete for ligand binding and may sequester TRAIL-R2 in non-functional complexes (reviewed in [25]).

It has been reported that hepatitis B virus core protein inhibits TRAIL induced apoptosis of hepatocytes by blocking TRAIL-R2 expression [26]. The protein levels of TRAIL-R1, TRAIL-R2, as well as the expression of decoy receptors, have been investigated in several HCC cell lines. However, these reports did not uncover so far any clear correlation between the basal profile of expression of the different TRAIL receptors and TRAIL resistance [27,28], pointing to a role of other molecular mechanisms that may significantly modulate TRAIL sensitivity in this context. Nevertheless, these and other studies confirm that the modulation of the expression levels of TRAIL-R1 and TRAIL-R2 achieved by several agents, including ionizing radiation, chemotherapy and histone deacetylase inhibitors, may provide a valuable approach to develop combined therapies to enhance the sensitivity of HCC cells to TRAIL induced apoptosis (reviewed in [29]). Interestingly, a first systematic report of the expression and cellular distribution of TRAIL-receptors in primary HCC suggests that loss of TRAIL receptors is a frequent feature of HCCs and correlates with a decreased survival of patients [30].

### 3.2. c-FLIP Proteins

Cellular FLICE Inhibitor Proteins (c-FLIPs) are a group of proteins that share structural and sequence homology with caspase-8 and caspase-10 but lack enzymatic activity. In particular, Like these caspases, they display the presence of Death Effector Domains (DEDs), which allow their interaction with FADD. Therefore, c-FLIPs can compete with caspase-8 and -10 for the recruitment to the DISC and may therefore impair caspase activation and the downstream apoptotic signalling. Several isoforms of cFLIP, arising from alternative splicing, are normally present in most cell types and may impair DISC activation through different molecular mechanisms (reviewed in [31]). The short isoform of cFLIP (cFLIP_S_) behaves as a pure inhibitor of procaspase-8 activation and Fas-induced apoptosis, as it contains only the DED domains, required for the DISC recruitment. The long isoform of cFLIP (cFLIP_L_) has two DED domains, and a caspase-like domain. Unlike the two caspases cFLIP lacks the cysteine residue in the active site and it is therefore catalytically inactive. Although, its heterodimerization with caspase-8 or caspase-10 may augment the latter’s enzymatic activity in some contexts (reviewed in [31]), at higher levels, it may displace initiator caspases from FADD and inhibit apoptotic signaling (reviewed in [32]). Importantly, aberrant up-regulation of cFLIP proteins has been shown to be present in a number of cancers and their down-regulation is sufficient to sensitise TRAIL-resistant tumour cell lines to apoptosis induction by TRAIL(reviewed in [33]). cFLIP_L_ is the most abundant isoform in many cancer cell lines, and its selective depletion is sufficient to enhance the proapoptotic signalling by TRAIL [34]. Interestingly, cFLIP_L_ is more expressed in human HCC tissues than in normal liver, and its protein levels are high in several HCC cell lines. Furthermore, also in this cellular context, c-FLIP downregulation enhances death receptor sensitivity [35], as further detailed in the next sections.

### 3.3. Bcl-2 Family

In many tumor cell lines TRAIL-induced apoptotic signalling requires the signal amplification through the mitochondria. Interestingly, the mitochondrial apoptosis pathway is mainly regulated by the balance of anti-apoptotic and pro-apoptotic members of the Bcl-2 family (an exhaustive recent review on the Bcl-2 family is provided in [36]). Bcl-2 overexpression protects neuroblastoma, glioblastoma and breast carcinoma cell lines from TRAIL induced apoptosis [37]. Importantly, an imbalance in the pro- and anti-apoptotic members of the Bcl-2 family is frequently reported in HCC (reviewed in [38]). Bcl-XL as well as Mcl-1 are aberrantly upregulated in a high percentage of HCCs [39,40]. Conversely, it has been clearly shown that the deletion of the pro-apoptotic family member Bax, is sufficient to confer complete resistance to TRAIL-induced apoptosis [41]. Interestingly, pro-apoptotic members of the family, such as Bax and Bcl-XS are downregulated in a subset of HCCs [42] suggesting that their deregulation may contribute also in this tumorigenic context to TRAIL resistance.

### 3.4. IAPs

Caspase activity is modulated by IAPs, which can bind executioner caspases a well the initiator caspase-9 impairing their activity. Mammalian homologous of IAPs, including cIAP1, cIAP2, XIAP and survivin have been associated to tumor development and cancer resistant to treatment in a wide range of human cancers [43]. Genetic targeting of XIAP, as well as IAP antagonists that mimic Smac (an endogenous inhibitor of IAPs), strongly enhances tumor cell sensitivity to TRAIL [44].

Nearly 90% of advanced HCCs express high levels of XIAP and in vitro studies suggest a correlation between metastasis, resistance to apoptosis and increased expression of XIAP [45]. Genome-wide analysis has recently identified cIAP1 as part of an amplicon recurrently amplified in a mouse model of liver cancer and in HCC tissue [46]. Similarly, survivin is also overexpressed in HCC cell lines and tissues [47] and it has been suggested that it might play an essential function in metastasis and therefore its expression may correlate with high risk of recurrence and poor prognosis [48].

### 3.5. NF-kappaB

The NF-κB pathway has been mainly associated to prosurvival programs, such as the transcriptional activation of cFLIP, IAPs and antiapoptotic Bcl2-family members (an exhaustive review on NF-κB is provided in [49]). Constitutive activity of the NF-κB pathway has been reported in certain tumors and may contribute to inhibit their apoptotic response [50]. Interestingly, genetic and pharmacological inhibition of the NF-κB signalling confers greater sensitivity to TRAIL in many cancer cells (reviewed in [29]).

The role of the NF-κB pathway in the modulation of HCC sensitivity to TRAIL is debated. Although some chemotherapy treatments enhance TRAIL sensitivity independently on NF-κB [27], evidence for the requirement of NF-κB inhibition for the execution of TRAIL-induced apoptosis has been provided [51] and interferon-α has been shown to trigger TRAIL sensitivity through the inhibition of NF-κB signalling cascade [52].

### 3.6. Tyrosine Kinases

It is well established that the signalling triggered by some receptor tyrosine kinases, such as the receptors for EGF, HGF and IGF-1, are aberrantly induced in human HCC [53]. Furthermore, the non receptor tyrosine kinase c-Src is costitutively iperactivated in hepatoma cell lines, where it accounts for their resistance to TRAIL-induced apoptosis [54]. Remarkably, Src inhibition synergizes with TRAIL only in cancer cell lines and not in primary hepatocytes. The constitutive activation of c-Src prevents caspase-8 activation [54]. Indeed Src has been previously shown to directly phosphorylate caspase-8 on Tyr380. As a consequence, caspase-8 processing and activation in response to Fas and TRAIL is strongly impaired and the apoptotic response is significantly blocked [55].

### 3.7. STAT Proteins

Signal transducer and activator of transcription (STAT) proteins are activated by tyrosine kinases in response to growth factors and cytokines. STAT3 is constitutively activated in HCC tissues and hepatoma cell lines through several mechanisms (reviewed in [56]). Importantly, abrogation of constitutive STAT3 activity significantly sensitizes hepatocellular carcinoma cell lines to TRAIL [57].

### 3.8. PI3K/Akt Pathway

The PI3K/Akt pathway is aberrantly regulated in HCC. PTEN expression is reduced in about 50% of HCCs and the selective inhibition of PTEN expression in hepatocytes results in HCC development in mice [58]. Interestingly, the pharmacological targeting of PI3K has been shown to be a valuable approach to sensitize HCC cell lines to TRAIL [59].

## 4. Development of TRAIL-Based Combined Therapeutic Approaches in HCC

Clinical trials with recombinant TRAIL have indicated that HCC might be resistant to TRAIL monotherapy [12]. For this reason, in the last ten years, several studies have been aimed to the development of novel approaches that may amplify TRAIL induced apoptosis signalling, in order to maximize the therapeutic potentiality of TRAIL or of TRAIL-R1 or TRAIL-R2 agonist antibodies in the treatment of hepatocarcinoma. The aim of these studies has been the identification of novel drugs that may selectively increase TRAIL sensitivity and therefore overcome the resistance of transformed hepatocytes towards TRAIL induced cytotoxicity (an extensive list of the compounds tested in combination with TRAIL in HCC cellular models has been provided in Table 2).

**Table 2:** TRAIL based combined therapies.

Type of Combined Therapy	Agent	Mechanism of Action	References
DNA Damage drugs	5-FU	downregulation of FLIP, upregulation of *TRAIL* receptors	[27]
	Cisplatin	downregulation of FLIP, upregulation of *TRAIL* receptors	[60]
	Etoposide	upregulation of Bax, increased release of cytochrome c and DIABLO	[61,62]
Inhibitors of target molecules	HDAC inhibitors (SAHA, valproic acid)	downregulation of FLIP, upregulation of *TRAIL* receptors	[63]
	protesome inhibitors (bortezomib)	downregulation of FLIP,upregulation of *TRAIL* receptors, suppression of Akt pathway	[64]
	Cyclooxygenase (COX)-2 inhibitors (NS398 and CAY10404)	up-regulation of TRAIL receptors, down-regulation of both survivin and AKT signaling	[65]
	ABT-263	inhibition of the Bcl-2 family	[66]
Kinase inhibitor	Genistein (isoflavone, tyrosine kinase inhibitor)	increasd cleavage of Bid, suppression of p38 MAPK signaling	[67,68]
	Quercitin (flavonoid, inhibitor of I-kappaB kinase)	downregulation of FLIP, upregulation of *TRAIL* receptors	[69]
	Flavopiridol (cyclin-dependent kinase)	upregulation of TRAIL receptors, down-regulation of survivin, FLIP and Bcl-xL	[70]
	Sorafenib* (multi-*kinase inhibitor*)*	downregulation of STAT3 phosphorylation, down-regulation of Mcl-1	[71]
	JNK inhibitor (AS601245, SP600125)	enhancer of caspase-8 activity and the downstream recruitment of the mitochondrial machinery	[72]
	Glycogen synthase kinase-3 inhibitors (lithium and SB-415286)	enhancer of caspase-8 activity and the downstream recruitment of the mitochondrial machinery	[73]
	Casein kinase 2 (emodin)	upregulation of TRAIL receptors	[74]
	Janus kinase 2 inhibitor (AG490)	inhibition of STAT3, XIAP and survivin	[75]
	Src-kinase inhibitor (PP2)	inhibition of caspase-8 activity	[54]
Natural Compound and Synthetic Drugs	Capsaicin	upregulation of *TRAIL* receptors	[76]
	Flavonoid and flavonoid-like chemical compound (Wogonin, 5, 7-dimethoxyflavone)	downregulation of FLIP, upregulation of *TRAIL* receptors	[77]
	Parthenolide	inhibition of STAT3,upregulation of *TRAIL* receptors	[78]
	Butein	NF-kappaB inactivation, upregulation of *TRAIL* receptors	[79]
	beta-Ionone	upregulation of *TRAIL* receptors	[80]
	Synthetic cannabinoid	upregulation of *TRAIL* receptors	[81]
	2-Phenyl-4-quinolone	upregulation of *TRAIL* receptors	[82]
	8-Chloroadenosine	upregulation of *TRAIL* receptors	[83]
	Quinacrine	downregulation of MCL-1, upregulation of *TRAIL* receptors	[84]
	Curcumin	ROS-mediated upregulation of TRAIL Receptors	[85]
	J7, a methyl jasmonate derivative	ROS-mediated upregulation of TRAIL Receptors	[86]
	Guggulsterone	ROS-mediated upregulation of TRAIL Receptors	[87]
	Peroxiredoxin I	ROS-mediated upregulation of TRAIL Receptors	[88]
	Sulforaphane	ROS-mediated upregulation of TRAIL Receptors	[89]
	Interferon-alpha	downregulation of Bcl-2, upregulation of *TRAIL* receptors and of Caspase-8, NFkappaB inhibition	[52]
	Celecoxib	downregulation of FLIP	[90]
	Melittin	activation of CaMKII-TAK1-JNK/p38, inhibition of IkappaBalpha kinase-NFkappaB.	[91]

### 4.1. DNA Damage Drugs

The first approach for the development of TRAIL based combined therapies in HCC cells lines has been based on the use of chemotherapeutic agents (Table 2). Yamanaka *et al.* showed for the first time that HCC cell lines were significantly sensitized to TRAIL-induced apoptosis by chemotherapeutic agents that induce DNA damage, in particular, doxorubicin (topoisomerase II inhibitor), camptothecin (topoisomerase I inhibitor), 5-fluorouracil (5-FU). However, the mechanism through which these drugs overcome tumor resistance to TRAIL-mediated apoptosis, has not been extensively investigated by the authors [27]. More recently, several studies have shown that chemotherapeutic agents such as cisplatin [28], and etoposide [61] are also effective in sensitizing HCC cells to TRAIL induced apoptosis. Furthermore, these studies provide also the molecular mechanisms underlying the ability of these drugs to enhance TRAIL sensitivity (Table 2). In particular, cisplatin and 5-FU trigger the downregulation of the antiapoptotic proteins cFLIP and the upregulation of TRAIL*-*R1 and TRAIL-R2 receptors and may efficiently sensitize HCC cells, but not normal hepatocytes to TRAIL-induced apoptosis [28,60]. Conversely the synergistic effect of TRAIL and etoposide, does not rely on the increase of the expression of TRAIL receptors, but rather is associated with the amplification of the mitochondrial signal pathway [61]. Interestingly these works demonstrated also that sensitization of HCC cells to TRAIL-induced apoptosis is largely independent of p53 status, a major player in DNA damage response.

### 4.2. Inhibitors of Target Molecules, Kinase Inhibitors, Natural Compounds and Synthetic Drugs

Additional to chemotherapeutic agents, different type of drugs that show synergy with TRAIL in HCC have been identified, and these include histone deacetylase (HDAC) inhibitors [92], proteasome inhibitors as bortezomib [64], kinase inhibitors, such as sorafenib [71] and natural compound (for more information see Table 2).

HDAC inhibitors, as well as proteasome inhibitors, sensitize HCC cells to TRAIL induced apoptosis mainly through the downregulation of cFLIP proteins without significant effects on primary human hepatocytes, showing a tumor cell-specific synergy [93,94].

Interestingly, the multiple kinase inhibitor, sorafenib (Nexavar), has been already shown to promote survival in patients with advanced HCC in large phase III studies and it is the first clinically approved drug for HCC [95]. Therefore the observation that sorafenib may trigger the sensitization of resistant HCC cells to TRAIL-induced apoptosis through STAT3 inhibition [71] or through Mcl-1 downregulation [59], point to this compound as an attractive drug for TRAIL combined therapeutic approaches. At present, three clinical trials, based on the combination between sorafenib and TRAIL receptor agonistic antibodies are ongoing on patients affected by hepatocellular carcinoma (Table 3). Other TRAIL clinical trials including patients with liver cancer are based on the combination with a receptor tyrosine kinase inhibitor (AMG 479, targeting the insulin-like growth factor receptor type 1) in the presence or not of conventional chemotherapy approaches (see Table 3).

### 4.3. siRNA Based Approaches

The increased knowledge of some of the molecular components of the apoptosis and survival pathways in HCC cell lines, has paved the way for the development of more specific agents that target crucial signaling components by selective siRNA (see Table 4). For example, since TRAIL resistance of different hepatocellular carcinoma cellular models has been clearly associated to the aberrantly enhanced expression of antiapoptotic proteins belonging to Bcl-2 and IAPs families, siRNA based strategies seeking to selectively antagonize the expression of these proteins have been successfully developed to sensitize hepatocarcinoma cells to TRAIL based cancer therapy [96].

**Table 3:** TRAIL based combined clinical trials.

Identifier	Cancer	TRAIL	Combined Treatment	Phase
NCT00712855	HCC	mapatumumab	sorafenib	I
NCT01258608	HCC	mapatumumab	sorafenib	II
NCT01033240	Advanced HCCLiver CancerHepatic CancerLiver Neoplasms	tigatuzumab (CS-1008)	sorafenib	II
NCT00819169	CRC *NSCLC*Locally AdvancedMetastatic Cancer Ovarian CancerPancreatic CancerSarcoma Solid Tumors	conatumumab (AMG655)	ganitumab (AMG 479)	II
NCT01327612	Advanced Solid TumorsCRCLocally AdvancedLymphomaMetastatic CancerNSCLC Solid Tumor	conatumumab (AMG655)	FOLFOX6 ganitumab (AMG 479) bevacizumab	II

**Table 4:** siRNA studies to enhance TRAIL sensitivity.

siRNAs Target	Reference
Gli2	[97]
COX2	[98]
DNA methyltransferases (DNMTs)	[99]
hTERT	[100]
Notch-1	[101]
Caveolin	[102]
XIAP	[103]
Survivin	[96]
Mcl-1	[104]

In conclusion, combined therapeutic approaches may be useful in the treatment of HCC as they contribute to the selective sensitization of transformed cells to the anti-tumoral activity of TRAIL. Moreover, although an extensive number of molecules and mechanisms have been shown to modulate the sensitivity to TRAIL-induced apoptosis in cancer cells [23,105], the general landscape is still quite confusing. As an example, many drugs seem to be toxic and to act in a very specific context. Therefore, the improvement of the knowledge underneath the molecular mechanisms involved in the balance between TRAIL resistance and TRAIL sensitization is urgently needed and a special effort should be aimed to identify new biomarkers that may allow the recognition of those patients who may profit from the therapeutic potential of this pathway without suffering from potential non-desired toxic effects.

## 5. ATM Kinase: A Novel Player in the Development of TRAIL Based Approaches for Cancer Therapy

### 5.1. ATM Kinase: An Essential Guarantee for Genomic Stability

The Ataxia Telangiectasia Mutated (ATM) has been originally identified as the product of a gene defective in a rare genetic disorder named Ataxia Telangiectasia (A-T), characterized among other features, by progressive cerebellar neurodegeneration leading to ataxia, dysfunctions of the immune system and higher incidence of lymphoma and leukaemia development. ATM is a key guardian of genomic stability. It is a serine treonine kinase activated very early in response to DNA damage essential to signal the presence of a DNA lesion and to prime the DNA damage response, including cell cycle arrest, repair and restore of the damaged chromatin or, in case the repair cannot be successfully carried out, the switch to the apoptotic response, to prevent in any case the replication of a cell with damaged DNA (reviewed in [106]). Therefore, it is not surprising that the deregulation of ATM activity has also been linked to several other pathological situations such as tumorigenesis, and metabolic syndromes including diabetes (reviewed in [107]).

The molecular mechanisms that ensure ATM kinase activity regulation and allow its induction in response to DNA damage and to other stresses have been largely investigated. ATM activity is tightly modulated in the cell through a complex of posttranslational modifications including phosphorylation and acetylation (reviewed in [107]). DNA damage triggers, among the other modifications, the autophosphorylation of ATM on Ser1981 [108], currently employed by the scientific community as a marker of ATM activation.

Upon activation ATM triggers the phosphorylation of several substrates, which in turn execute the DNA damage response, allowing cell cycle arrest and repair, or apoptosis. As an example, ATM mediates the downstream phosphorylation and activation of other checkpoint kinases, such as Chk2, as well as the phosphorylation of the central tumor suppressor gene p53, both required for a functional DNA damage response (reviewed in [109]). Recently, several proteomic approaches identified about 1,000 ATM substrates in response to DNA damage, strongly supporting the central role of ATM activity in this signalling cascade [110].

Overall, the role of ATM in the execution of the DNA damage response and in the preservation of genomic stability pointed to ATM as a tumor suppressor gene. Consistently, A–T patients display a significantly higher predisposition to the development of lymphoma and leukaemia. Moreover Atm heterozygous carriers are at increased risk of cancer, particularly breast cancer (reviewed in [109]).

Since ATM has a key function in the DNA damage response, the value of ATM kinase inhibition as a novel tool to sensitize tumors to DNA damaging therapeutic approaches such as radiotherapy and chemotherapy has been largely investigated [111]. Recently, it has been shown that ATM kinase activity may exert positive or negative effects in cancer therapy [112] pointing to the requirement for studies that further elucidate the possible connections between ATM activation and cancer markers.

### 5.2. Role of ATM in Liver Homeostasis and Carcinogenesis

The role of ATM kinase in liver development and function has not been fully elucidated, so far. However, recently the contribution of ATM deficiency to the carcinogenic response of hepatocytes to diethylnitrosamine (DEN) has been investigated [113]. Surprisingly, hepatocarcinogesis is significantly delayed or abrogated, in ATM-defective mice. The authors show that DEN in the absence of ATM kinase triggers a strong induction of p53, most likely as a consequence of ATR and Chk1 upregulation, and suggest that p53 induction renders Atm-deficient mice refractory to hepatocarcinogenesis. In response to DEN treatment, livers from Atm KO mice display also impairment of cyclin A and D1 induction and conversely, increased caspase-3 activation as well as increased expression of markers of senescence, such as β-galactosidase and Cxcl-1, compared to wt. Since ATM modulates telomere function, the authors also evaluated the level of TERT expression in wt and in Atm^−/−^ livers before and after DEN treatment. The degree of TERT expression was significantly higher in treated livers derived from wt animals compared to the Atm ko ones, suggesting that the activation of telomerase in this context may provide a mechanism to escape cellular senescence. To discern whether TERT induction parallels with the normal hepatocyte proliferative activity, wt and ko mice were subjected to two thirds partial hepatectomy. There were no significant differences in the hepatocyte proliferation between wt and ko mice [113]. This observation is in contrast with a previous report where evidence for a role of ATM in hepatocyte survival and in liver regeneration has been provided [114]. The authors described a strong increase in ATM protein level expression in response to liver regeneration triggered by partial hepatectomy, which parallels with an increase in DNA synthesis, p53 phosphorylation on Ser23 (equivalent to human Ser20), induction of cyclin A and of p21. Conversely hepatocytes derived from Atm^−/−^ mice show a defective induction of cyclin A and of DNA synthesis, delayed p53 phosphorylationon Ser23 and increased and sustained p21 expression, which may account for the defective cell cycle progression [114]. Overall, despite some discrepancies between the two studies, these data suggest that ATM kinase activity may be significantly induced in hepatocytes in response to stresses triggered by hepatectomy or by cancerogenic agents and in turn, may modulate the balance between proliferation, cell death and senescence.

### 5.3. Role of ATM in TRAIL Signalling

It has been shown that the expression of TRAIL death receptor TRAIL-R2 is transcriptionally modulated in some circumstances by DNA damage through p53 and that ATM deficient lymphoblastoid cells fail to up-regulate TRAIL-R2 in response to ionizing radiation, suggesting that ATM activity might contribute to promote TRAIL sensitivity [115].

**Figure 1:**
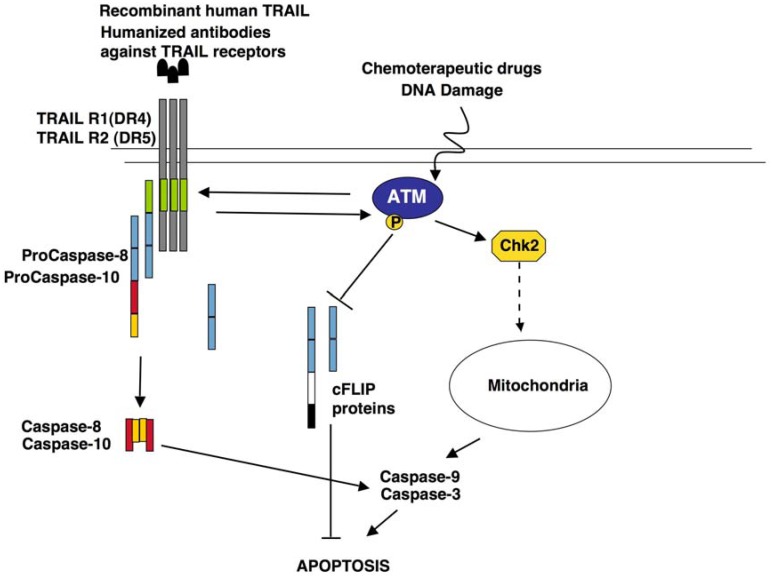
ATM kinase activity modulates TRAIL sensitivity. DNA damaging agents trigger ATM kinase activity which in turn modulates TRAIL sensitivity through three mechanisms: (1) ATM activity promotes TRAIL R2 expression [115]; (2) TRAIL-R1 and TRAIL-R2 stimulation, further promote ATM catalytic activity [117,118] and triggers downstream Chk2 activation, which triggers Caspases activation through the mitochondria pathway [117]; (3) ATM activity directly down regulates cFLIP proteins levels [118]. Overall these signalling pathways identify ATM as a central modulator of TRAIL sensitivity triggered by DNA damaging chemotherapeutic drugs.

Recently, evidence for the activation of ATM kinase and of other components of the DNA damage response following death receptor stimulation, has been provided (Figure 1). Fas stimulation triggered the typical ATM-dependent phosphorylation cascade. Indeed, ATM was phosphorylated on its autophosphorylating activating site *i.e.*, Ser1981 [108], and p53, Chk2 and H2AX became phosphorylated at Ser15, Thr68 and Ser139 respectively. These data indicate that Fas stimulation results in ATM activation [116]. Similarly to Fas, TRAIL induces a rapid activation of the DNA damage response allowing ATM, DNA-PK, Chk2 and histone H2AX phosphorylation [117]. Importantly, the inhibition of ATM or of Chk2 activity results in reduced Fas and TRAIL sensitivity, pointing to the DNA damage response signaling cascade as a modulator of death receptor function (Figure 1) [116,117,118].

The apoptotic response triggered by stimulation of death receptors such as Fas and TRAIL is significantly modulated by the expression of c-FLIP proteins, which are indeed major resistance factors and critical anti-apoptotic regulators in this context. It is well established that c-FLIP proteins are aberrantly enhanced in many tumors and their up-regulation has been described as a molecular mechanism that significantly contribute to tumorigenesis. Furthermore, c-FLIP proteins induction impairs in many cellular contexts death receptor sensitivity, providing a molecular mechanism for the restriction of the sensitivity to TRAIL in therapeutic approaches. Consistently, several combined therapies developed to enhance TRAIL sensitivity, have been shown to impinge on FLIP-protein levels through several molecular mechanisms that are still largely uncovered [62,119,120,121]. We have recently identified ATM kinase as a novel modulator of Fas and TRAIL sensitivity (Figure 1) [116,118]. Lymphoblastoid cell lines derived from A-T patients are significantly resistant to Fas and TRAIL induced apoptosis, which can be rescued by reconstitution of ATM expression. Consistently, genetic and pharmacological inhibition of ATM expression is sufficient to trigger Fas and TRAIL resistance in a panel of wt cell lines, including Hodgkin lymphoma cell lines and hepatocellullar carcinoma cell lines. We have shown that ATM activity impinges on the control of the expression of c-FLIP_L_ and c-FLIP_S_ proteins. The inhibition of ATM kinase activity is sufficient to enhance the expression of c-FLIP proteins, which is responsible for death receptor resistance [116,118]. Importantly, we could show that chemotherapy agents that trigger DNA damage may mediate c-FLIP protein downregulation and subsequently sensitize cells to death receptor induced apoptosis, through an ATM-dependent mechanism. We could show that proteasome inhibition leads to cFLIP_S_ accumulation independently on ATM expression and activity, while the levels of cFLIP_L_ protein increases selectively in ATM proficient cells. Moreover, DNA-damage-induced activation of ATM selectively renders cFLIP_L_ but not cFLIP_S_, more susceptible to ubiquitination and degradation via the ubiquitin-proteasome pathway. In conclusion, ATM activity contributes to DNA damage-induced sensitisation to TRAIL-induced apoptosis although it triggers the down-regulation of cFLIP_L_ and cFLIP_S_ by DNA-damaging agents through different mechanisms [118]. We and others suggest that in several cancer contexts, among which HCC [116,117,118], ATM expression and status might play a role in the efficacy of chemotherapy agents that trigger DNA damage as ehancers of TRAIL sensitivity. Conversely, in melanoma cell lines, ATM has been identified as a negative modulator of TRAIL sensitivity [122], suggesting that different contexts may account for different responses. In this, regard it is also interesting to consider that TRAIL stimulation may also contribute to DNA damage supporting the idea that in some conditions it may synergize with ATM inhibition [123]. Recent findings that autophagy may play a role in chemoresistance through the enhancement of the ATM-dependent DNA repair [124], and may contribute to TRAIL resistance counteracting the induction of apoptosis [125,126], suggest that the interplay between ATM and TRAIL signalling may be modulated at multiple levels.

Further studies will allow to deeply evaluate whether and in which contexts ATM expression and activity may represent a marker to predict the efficacy of chemotherapy agents as valuable approaches for combined strategies to enhance cancer cell sensitivity to TRAIL.

## 6. Conclusions

HCC is still characterized by poor prognosis and the development of more effective therapeutic strategies is a major issue in the field. In this regard, the employment of TRAIL has been shown to provide a novel promising approach because of its specificity for cancer cells and of its lack of toxicity for healthy cells. However, clinical trials uncover the occurrence of several molecular mechanisms that trigger TRAIL resistance in several tumors among which HCC, pointing to the relevance of studies aimed to the identification of combined therapeutic approaches that may overcome this resistance and improve the efficacy of TRAIL-based therapy. In this regard, DNA damaging agents, which are largely used in chemotherapy, have been shown to be effective as they significantly enhance TRAIL sensitivity mainly through the downregulation of the cFLIP proteins, which are master modulators of death receptor induced apoptosis. The identification of ATM kinase as novel modulator of cFLIP proteins in response to several chemotherapeutic agents, point to ATM as a central modulator of TRAIL sensitivity (Figure 1). In this regard, the analysis of ATM expression and activity, using immunohistochemistry approaches with specific antibodies may allow to predict the ability of DNA damaging agents to trigger ATM activation and FLIP downregulation in those tumors where TRAIL resistance has been clearly linked to FLIP proteins upregulation. Future studies will clarify whether and in which conditions ATM may represent a putative marker for the screening and the identification of those tumors which may be successfully targeted by the combination of TRAIL and DNA damage drugs (discusses also in [127]). This finding may contribute to the development of more personalized and therefore effective targeted therapies.
